# Racial and socioeconomic disparity associates with differences in cardiac DNA methylation among men with end-stage heart failure

**DOI:** 10.1152/ajpheart.00036.2021

**Published:** 2021-03-26

**Authors:** Mark E. Pepin, Chae-Myeong Ha, Luke A. Potter, Sayan Bakshi, Joseph P. Barchue, Ayman Haj Asaad, Steven M. Pogwizd, Salpy V. Pamboukian, Bertha A. Hidalgo, Selwyn M. Vickers, Adam R. Wende

**Affiliations:** ^1^Division of Molecular and Cellular Pathology, Department of Pathology, University of Alabama at Birmingham, Birmingham, Alabama; ^2^Institute for Experimental Cardiology, Heidelberg University Hospital, Heidelberg, Germany; ^3^Division of Cardiovascular Medicine, Department of Medicine, University of Alabama at Birmingham, Birmingham, Alabama; ^4^Department of Epidemiology, School of Public Health, University of Alabama at Birmingham, Birmingham, Alabama; ^5^Office of the Dean and Senior Vice President For Medicine, School of Medicine, University of Alabama at Birmingham, Birmingham, Alabama

**Keywords:** DNA methylation, epigenetics, gene-environment interactions, heart failure, structural racism

## Abstract

Heart failure (HF) is a multifactorial syndrome that remains a leading cause of worldwide morbidity. Despite its high prevalence, only half of patients with HF respond to guideline-directed medical management, prompting therapeutic efforts to confront the molecular underpinnings of its heterogeneity. In the current study, we examined epigenetics as a yet unexplored source of heterogeneity among patients with end-stage HF. Specifically, a multicohort-based study was designed to quantify cardiac genome-wide cytosine-p-guanine (CpG) methylation of cardiac biopsies from male patients undergoing left ventricular assist device (LVAD) implantation. In both pilot (*n* = 11) and testing (*n* = 31) cohorts, unsupervised multidimensional scaling of genome-wide myocardial DNA methylation exhibited a bimodal distribution of CpG methylation found largely to occur in the promoter regions of metabolic genes. Among the available patient attributes, only categorical self-identified patient race could delineate this methylation signature, with African American (AA) and Caucasian American (CA) samples clustering separately. Because race is a social construct, and thus a poor proxy of human physiology, extensive review of medical records was conducted, but ultimately failed to identify covariates of race at the time of LVAD surgery. By contrast, retrospective analysis exposed a higher all-cause mortality among AA (56.3%) relative to CA (16.7%) patients at 2 yr following LVAD placement (*P* = 0.03). Geocoding-based approximation of patient demographics uncovered disparities in income levels among AA relative to CA patients. Although additional studies are needed, the current analysis implicates cardiac DNA methylation as a previously unrecognized indicator of socioeconomic disparity in human heart failure outcomes.

**NEW & NOTEWORTHY** A bimodal signature of cardiac DNA methylation in heart failure corresponds with racial differences in all-cause mortality following mechanical circulatory support. Racial differences in promoter methylation disproportionately affect metabolic signaling pathways. Socioeconomic factors are associated with racial differences in the cardiac methylome among men with end-stage heart failure.

Listen to this article’s corresponding podcast at https://ajpheart.podbean.com/e/racial-socioeconomic-determinants-of-the-cardiac-epigenome/.

## INTRODUCTION

Heart failure (HF) is the clinical end point of many chronic cardiovascular and metabolic diseases. Despite its multiple origins, HF is typically treated as a single disease, where pharmacologic management addresses the hemodynamic consequences of pump failure often independent of its underlying cause(s) (1). Guideline-directed management of HF has improved mortality following hospitalization (2); however, HF is still widely considered a chronic and progressive syndrome with increasing worldwide prevalence (3). Identifying the molecular origins of its pathogenesis has thus become a central focus of investigation in pursuit of novel diagnostic, prognostic, and therapeutic tools for HF.

In the search for precision-based treatments, studies have identified a host of de novo and inherited genetic mutations that predispose individuals to cardiomyopathy (4). Since the advent of sequencing technologies, over 100 familial cardiomyopathies have been found to involve structural, electrochemical, and metabolic genes (5, 6). In total, genetic contributions are estimated among 30% of HF cases (7). However, causal gene variants are accompanied by both variable penetrance and phenotypic heterogeneity, exhibiting an array of cardiac manifestations and clinical outcomes (5). Even single-gene familial cardiomyopathies display a broad clinical heterogeneity that cannot be explained by genetics alone (8).

Environmental influences have subsequently been identified as major determinants of HF severity and pathogenesis (9). Known risk factors for HF include chronic diseases such as coronary artery disease (10), hypertension (11), and diabetes mellitus (12). Other risks entail behavioral and environmental factors, including diet (13), exercise (14), and even chronic exposure to air pollutants (15). Although still poorly understood, a complex interplay is theorized to exist between genetic susceptibility and environmental triggers (16).

As indirect mechanisms of gene regulation, epigenetic influences provide a molecular framework for gene-environment interactions in HF. Epigenetics is a field that encompasses biochemical modifications directly to the DNA base-pair sequence (e.g., 5′ cytosine methylation), or those which affect auxiliary structures such as histone proteins. Although many epigenetic mechanisms serve crucial roles in the pathogenesis of heart disease (17), our laboratory has identified a pathological program of cardiac DNA methylation in human HF that encodes both disease-wide and etiology-specific signatures of metabolic gene expression (18, 19). No studies have yet identified the clinical qualities, singular or composite, that are most precisely represented by the cardiac methylome.

Therefore, in the current study we assess the usefulness of cardiac DNA methylation as a molecular signature of HF clinical diversity. Using multicohort-based unsupervised clustering analysis of cardiac genome-wide DNA methylation, we uncover a bimodal epigenetic signature that only categorical patient race could delineate. Geocoding-based analysis of patient demographics exposes socioeconomic underpinnings of racial disparity, which is not documented in medical records. This observational study therefore provides preliminary evidence that cardiac DNA methylation describes socioeconomic and racial differences in end-stage human heart failure.

## METHODS

### Ethics Statement Regarding Use of Human Tissue

All human studies were approved by the Institutional Review Board at the University of Alabama at Birmingham. A waiver of informed consent was provided to the UAB Tissue Bank for the procurement of tissue biopsies during the placement of left ventricular assist devices (LVAD), since procurement and processing of these tissues constitute a routine part of the surgical procedure. De-identified patient demographics, health information, and all-cause mortality were also obtained.

### Data Availability and Open-Sourced Bioinformatic Analysis

Raw and processed files for RNA-sequencing and DNA methylation analyses are made publicly available on the NCBI GEO database for both pilot (GSE109097) and testing (GSE164197) cohorts. A detailed description of the bioinformatic workflow, including all coding scripts and quality control metrics, are provided as an open-source *GitHub* repository: https://mepepin.github.io/Racial-Differences-in-the-Cardiac-Epigenome/.

### Cardiac Tissue Biopsy Selection and Data Procurement

Human samples of apical left ventricle were collected from patients with HF who underwent LVAD implantation surgery at the University of Alabama at Birmingham over a 10-yr span. Only endomyocardial sections of the cardiac apex were used in the analysis to control for regional heterogeneity in cardiac physiology, as has been previously reported (20). In both the pilot and testing cohorts, biopsies from male subjects were chosen with ages ranging from 49 to 70 yr. All patients with HF were considered end-stage (NYHA Class IV) and exhibited severe systolic dysfunction consistent with HF with reduced ejection fraction (HFrEF) based on bedside echocardiogram.

### Genome-Wide DNA Methylation Analysis

For both cohorts, cardiac DNA was extracted from whole-tissue LVAD samples using the DNeasy Blood and Tissue kit (Qiagen Inc., Hilden, Germany). For each assay, 500 ng of DNA was bisulfite-treated with the EZ DNA Methylation Kit (Zymo, Irvine, CA) before amplification, hybridization, and imaging standard to the Illumina protocol. To quantify cardiac DNA methylation, we used both Illumina Beadchip Human Methylation 450 K (methyl450K) and MethylationEPIC array platforms for the pilot and testing cohorts, respectively. The methyl450K array used for the pilot cohort interrogates 485,577 Cytosine-p-Guanine (CpG) sites and covers >99% of the RefSeq genes with an average of 17 CpG sites per reference gene and this data was previously analyzed and published in the context of ischemic heart failure (19). By comparison, the Illumina Human Methylation EPIC array for the subsequent testing cohort interrogates 865,859 CpG sites spanning the promoter, coding, and intergenic regions of all known protein-coding genes (Supplemental Fig. S1; all Supplementary material is available at https://doi.org/10.5281/zenodo.4657706), including >90% of the CpG probes used by the Human Methylation 450 K array.

**Figure 1.**
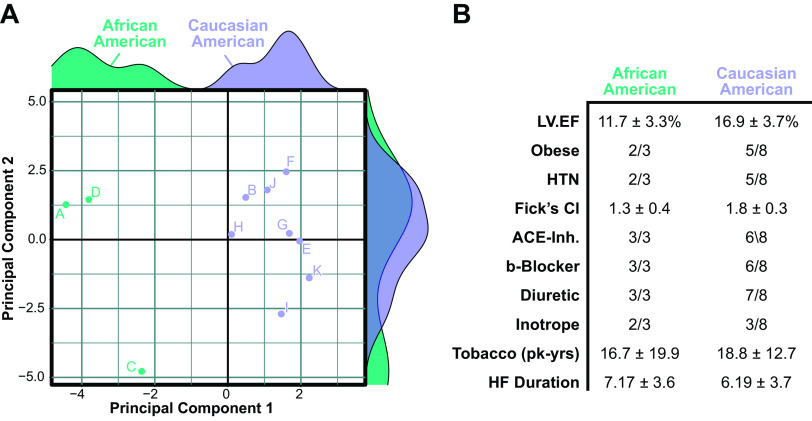
Pilot analysis of cardiac DNA methylation. *A*: multidimensional scaling (MDS) of top 10,000 cytosine-p-guanine (CpG) probes within the Illumina Human Methylation 450K array performed on cardiac left ventricle samples from patients with end-stage heart failure. The two principal components that account for the largest variance in DNA methylation were used to generate a scatterplot, flanked by density plots of each principal component. *B*: patient characteristics of cardiac samples used in the analysis. ACE-Inh, angiotensin-converting enzyme inhibitor; HF, heart failure; HTN, hypertension; LVEF left ventricular ejection fraction.

The methylation array data were then used to quantify the proportion of methylation at each CpG locus on a probe-wise basis using the R package *minfi* (1.36.0). Briefly, array intensity data (“.idat” files) generated via iScan were preprocessed and normalized using subset-quantile within array normalization (SWAN) which corrected for technical differences between the Type I and Type II array designs (21). Total (methylated + unmethylated) signal intensity for each probe was weighed against the background signal via negative control probes to provide a statistical (*P* value) detection threshold; using this method (Supplemental Fig. S2), a single outlier (LVAD054) was identified as having insufficient signal reliability owing to its low signal-to-background intensity relative to all other samples; for this reason, it was removed from subsequent analyses. Once data were assessed for signal quality, stratified quantile normalization was applied to both methylated and unmethylated signals across all samples, as recommended for single-source tissues (22). Differential methylation analysis was done by fitting probe-wise linear models to the normalized log-ratios, followed by an empirical Bayesian shrinkage of probe-wise sample variance (23).

### RNA Sequencing Analysis

RNA sequencing analysis was performed as previously outlined (19), with detailed methods available as an online supplement. Briefly, RNA was isolated using the RNeasy Fibrous Tissue Mini Kit (Qiagen Inc., Hilden, Germany) and validated to ensure RNA quality [RNA Integrity Numbers (RINs) > 7]. High-throughput mRNA-sequencing was executed via Illumina HiSeq2000 for paired-end 2× 100 bp sequencing runs. Adapters and low-quality (PHRED < 20) sequences were trimmed from reads files using Trim Galore (0.5.0), followed by alignment to the human genome (GRCh38.p12) via STAR (2.5.3a). Differential gene expression was then computed by categorical race using DESeq2 (1.30.0). Dispersion estimates were calculated via maximum likelihood (24), followed by gene-wise dispersion using empirical Bayes method. Differential gene expression is reported as a log_2_(fold-difference) from quantile-normalized read counts among African American (AA) and Caucasian American (CA) patients via the Wald test, with statistical significance assessed by Benjamini–Hochberg (BH) *P*-value correction (Supplemental Table S1).

### Statistics

For all pairwise comparisons, the Shapiro–Wilk test for normality was performed to determine the most appropriate statistical test. All patient factors exhibiting a parametric distribution (age, HF duration) were evaluated using Student’s *t* test with Benjamini–Hochberg adjustment; otherwise, a Mann–Whitney test was used. All data are reported as means ± standard deviation unless otherwise specified.

## RESULTS

### Pilot Cohort Analysis Uncovered a Racial Pattern of Cardiac DNA Methylation

The current study originated from the initial analysis of a cohort of patients with ischemic (*n* = 5) and nonischemic (*n* = 6) end-stage HF. Although we have previously reported that a patient’s history of ischemic heart diseases is most responsible for global differences in cardiac DNA methylation within this cohort (19), it was noted here that many of the CpG probes on the array exhibited only subtle differences in methylation intensity. We thus performed a follow-up analysis using unsupervised deconvolution via multidimensional scaling (MDS) from the 10,000 most-variable probes. Plotting the two eigenvectors responsible for the greatest proportion of sample variance revealed a bimodal distribution; it was noted from this that three samples (A, C, and D) clustered separately from the others (Fig. 1*A*). Overlaying patient information from electronic health records identified race as the only attribute that could delineate this MDS-based partitioning (Fig. 1*B*).

### Patient Characteristics of Testing Cohort

**Figure 2.**
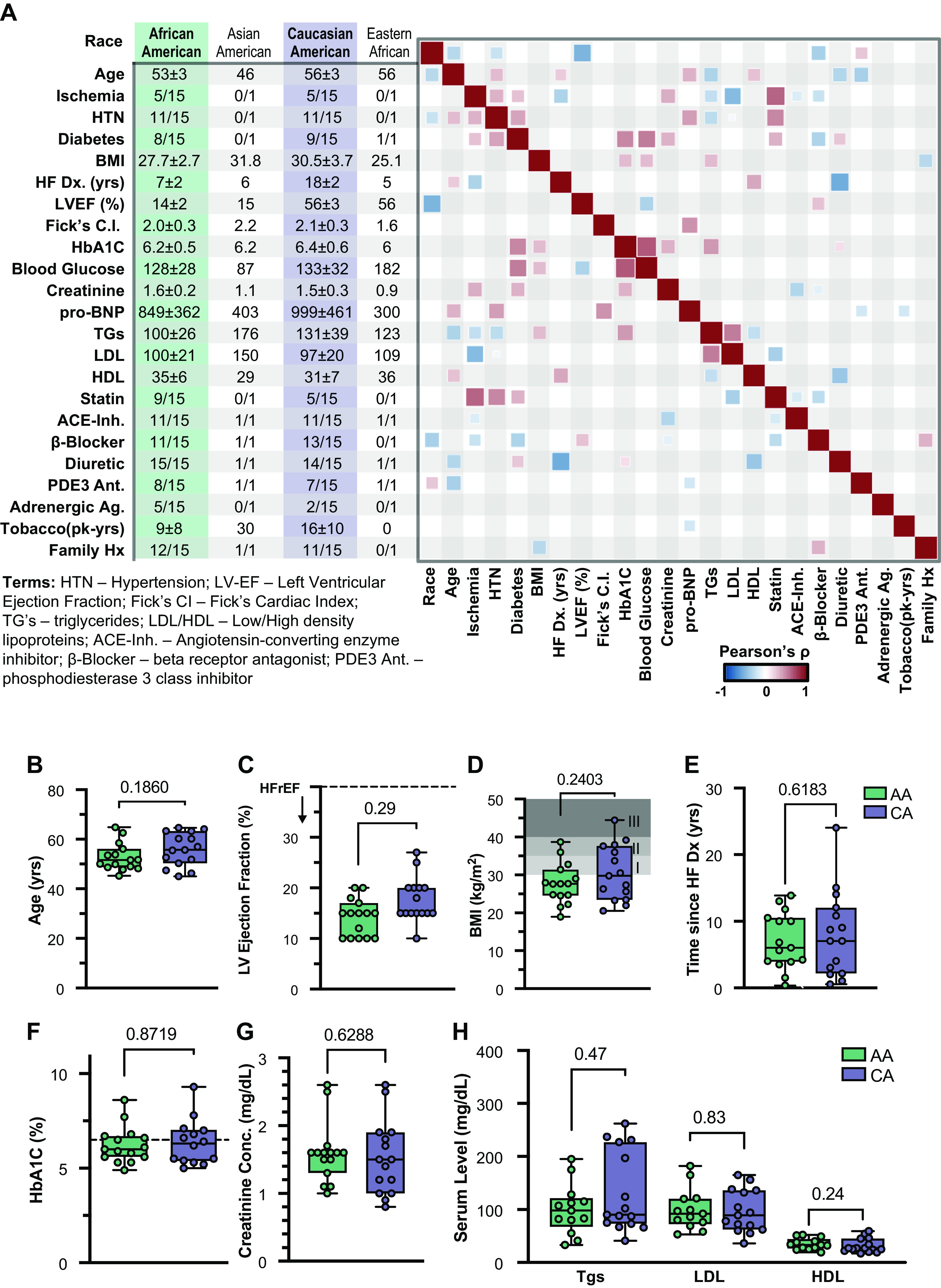
Patient characteristics of testing cohort. *A*: patient demographics and health information was obtained from the electronic health records (EHR), reporting either the means ± 95% CI or number of patients with a characteristic relative to number of patients categorized according to each race. Correlation heatmap performed on EHR data using chi-squared statistic, showing only direct (red) or inverse (blue) correlations that exceed a significance threshold of *P* = 0.05. Pairwise comparisons between African American (AA) and Caucasian American (CA) subjects were performed using Student’s *t* test statistic by patient age (*B***)**; LV ejection fraction (*C*); BMI (kg/m^2^), with Classes I–III obesity ranges illustrated using shading (*D*); duration of heart failure, measured as time from diagnosis to date of left ventricular assist device (LVAD) procedure (*E*); glycated hemoglobin A1C concentrations (HbA1C%) (*F*); creatinine concentrations on admission for LVAD (*G*); and serum circulating triglycerides, low-density lipoprotein (LDL), and high-density lipoprotein (HDL) levels (mg/dL) (*H*). All pair-wise comparisons were performed using unpaired two-tailed Student’s *t* tests.

Owing to its small sample size and limited medical documentation, the initial observations from our pilot analysis prompted a larger follow-up study of 32 LVAD patients with detailed medical history. Cardiac biopsies from patients with HF comprising equal numbers of African American (AA) and age-matched Caucasian American (CA) were collected for analysis (*n* = 15). Two additional samples were obtained from patients who identified as U.S. immigrants, both a first-generation eastern African and a second-generation eastern Asian, leading to the total of 32 samples; however, one sample was removed owing to concerns of poor analytic quality (see Expanded Methods). More extensive curation of medical records was performed to understand potential confounding effects of patient comorbidities and HF treatment regimens (Fig. 2*A*), followed by a pairwise comparison to identify any significant differences (Fig. 2, *B*–*H*). Although a few characteristics appeared to correlate with race, no significant differences were present by pairwise comparison upon correcting for multiple comparisons.

### Patient Outcomes

**Figure 3.**
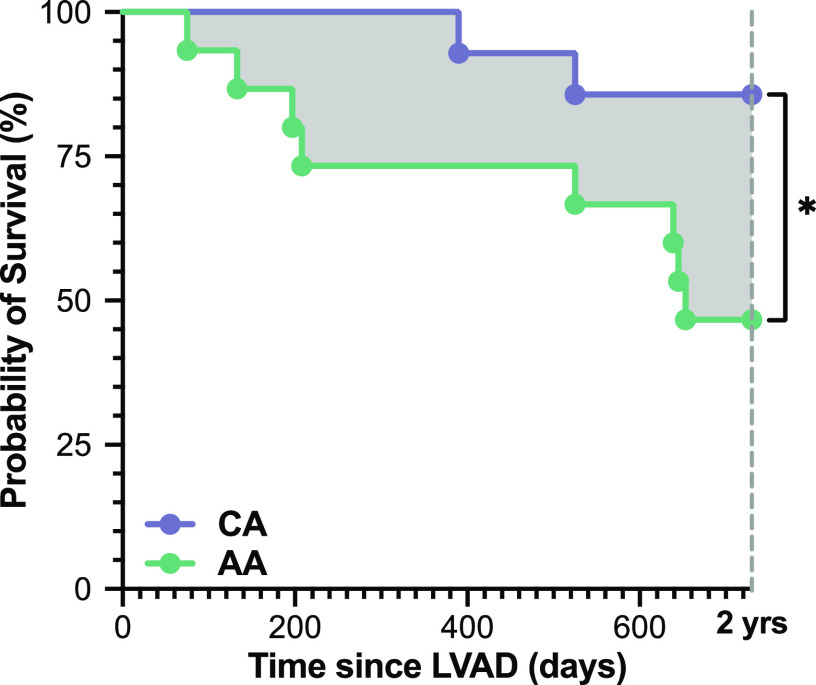
Racial difference in post-left ventricular assist device (LVAD) survival. Retrospective analysis of patient survival according to categorical patient race during the 2 yr following surgical implantation of a LVAD for circulatory support. Statistical comparison was made using a log-rank (Mantel-Cox) test (*P = 0.03). One Caucasian American (CA) subject was lost to follow-up during the 2 yr, and was thus removed from the comparison.

To determine whether the patient cohort under investigation displayed differing clinical outcomes in accordance with patient race, we performed a retrospective analysis of 2-yr all-cause mortality following LVAD implantation in the 31 subjects (Fig. 3). Although limited by sample size, this preliminary comparison revealed a significantly higher mortality in AA (8/15) relative to CA (2/14) subjects over the subsequent 2 yr (*P* = 0.033), though there were no racial differences in the transplantation rate (2 AA, 2 CA). This observation is consistent with prior reports of poorer outcomes among underrepresented minorities following LVAD implantation (25).

### Unbiased Analysis Revealed a Signature of Racial Bias in the Cardiac Epigenome

**Figure 4.**
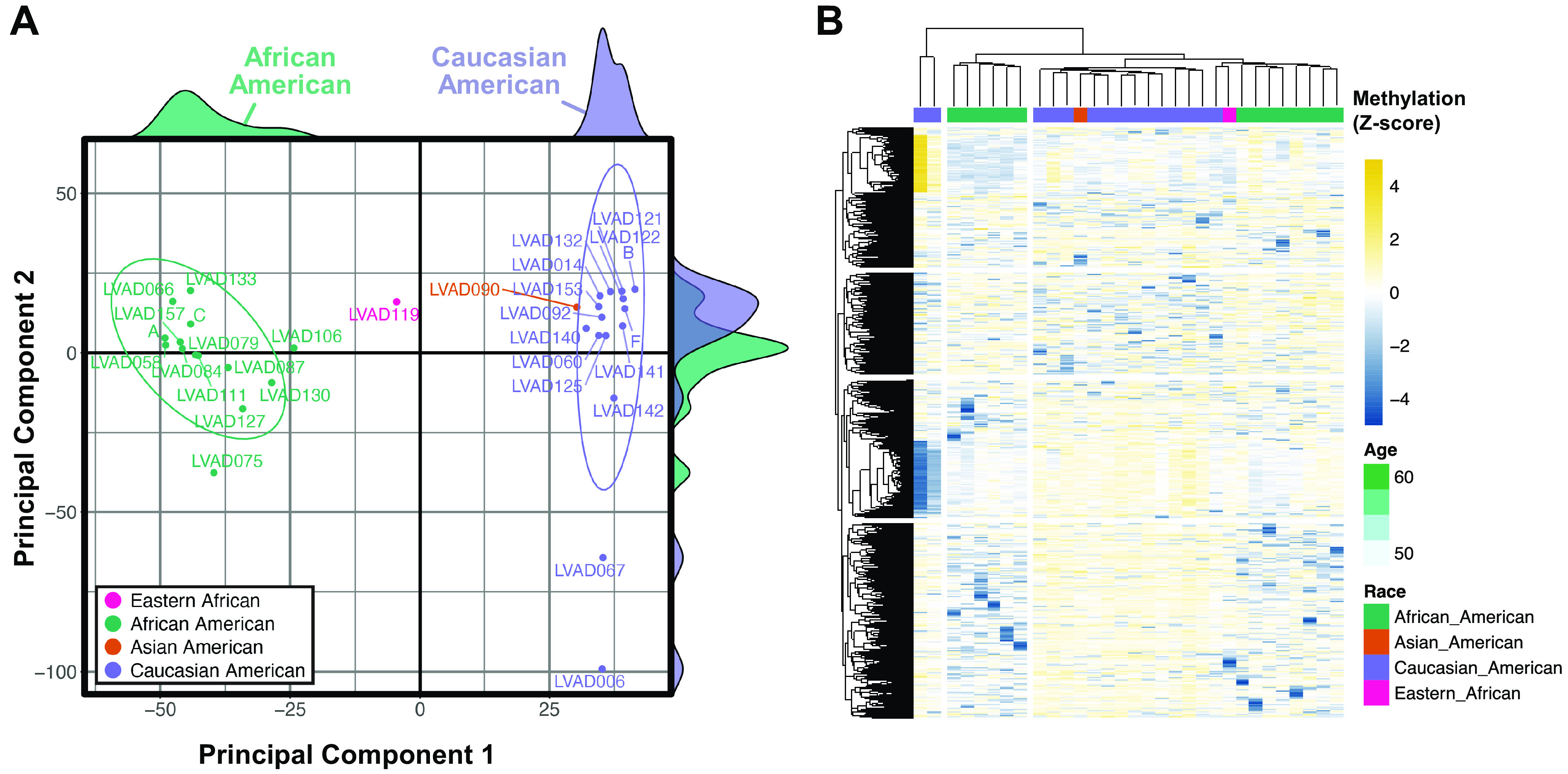
Unsupervised analysis of cardiac genome-wide DNA methylation in testing cohort. *A*: multidimensional scaling (MDS) plot of top 10,000 cytosine-p-guanine (CpG) probes within the Illumina Human Methylation EPIC array performed on cardiac left ventricle samples from patients with end-stage heart failure. The two principal components that account for the largest variance in DNA methylation were used to generate a scatterplot, flanked by density plots for each principal component denoting samples from African American (AA) and Caucasian American (CA) patients. *B***:** putative confounding of single-nucleotide polymorphisms and gene variants were computationally identified using MethyltoSNP, with methylation intensity of CpG probes clustered using Ward’s minimum variance method (ward.D2) of hierarchical clustering and heatmap visualization.

To replicate our preliminary observations of DNA methylation in the testing cohort, cardiac DNA methylation analysis was again performed using the 31 cardiac ventricular biopsies. To authenticate our initial observations using this larger cohort, unsupervised clustering of the 10,000 most-variable CpG probes was again performed from EPIC array data (Fig. 4*A*). Bimodal separation among samples reemerged and remained visible among the 500,000 most-variable CpG probes (Supplemental Fig. S3). As before, self-identified race was the only patient characteristic from medical records that could demarcate this clustering. Taken together, these observations suggest that epigenetic programs differ by race in the failing heart.

Owing to the reported influence of genetic polymorphisms on array-based quantification of differential methylation analyses (26–28), we sought to identify whether known and/or novel single-nucleotide polymorphisms (SNPs) were confounding our analysis of CpG sites interrogated by the EPIC array. We initially used the *dbSNP* database (29) to identify CpG probes that contain known polymorphic sites and regions; this resulted in annotation of 30,435 CpG probes; removing these did not eliminate our observed racial separation on MDS. Nevertheless, it was posited that unknown genetic dissimilarities could confound the epigenomic analysis. A computational method of “gap hunting” was used to infer β-value signals reflective of minor allele frequencies (30). From this, 1,294 CpG probes were identified as likely influenced by proximal SNPs and/or genetic variants (Supplemental Fig. S4); among these, 1,076 (83%) have already been identified as SNPs by the *dbSNP* database. Hierarchical clustering of normalized beta values among these probes demonstrated modest racial differences in these SNP-containing CpG probes between AA and CA samples (Fig. 4*B*); however, clustering was largely driven by two CA samples. Although all putative SNP sites were removed from the data set before subsequent analysis, these observations support that the race-based signature of cardiac CpG methylation is not confounded by genetic background.

### Cardiac Differential Methylation of African and Caucasian American Failing Hearts

**Figure 5.**
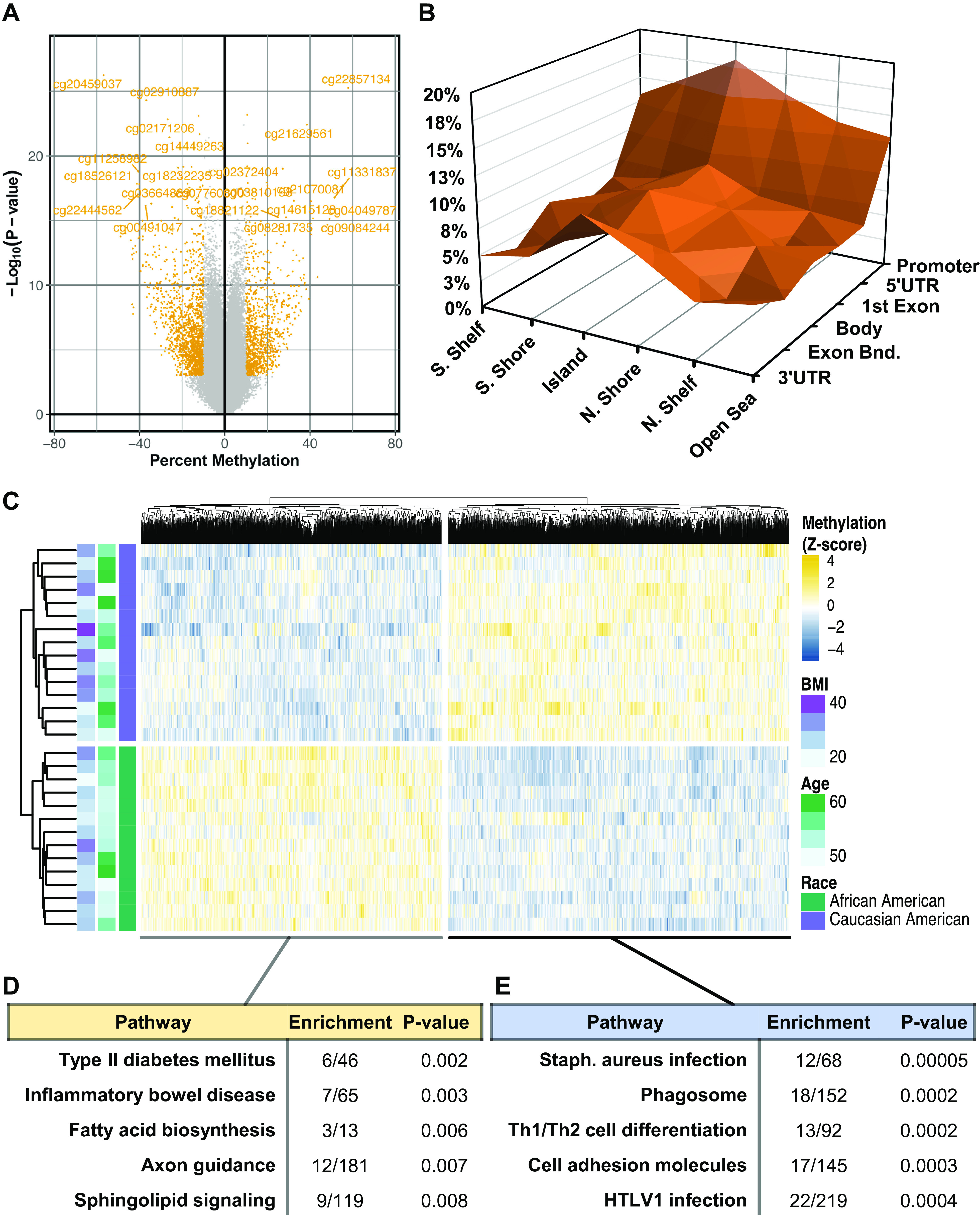
Racial differences in cardiac DNA methylation. *A*: volcano plot illustrating the robustness of cytosine-p-guanine (CpG) methylation differences, plotting (−log_10_[*P* value]) as a function of percent difference in methylation between African American (AA) and Caucasian American (CA) samples. Highlighted in yellow are CpG probes exceeding |10% Methylation| with *P* < 0.01. *B*: three-dimensional contour plot of differentially methylated CpG-probes (DMCs) categorized according to their presence within genomic (Promoter, 5′ UTR, body, exon-intron boundary, or 3′ UTR). *C*: Hierarchical clustering and heatmap visualization of DMCs; samples were annotated with categorical patient race, BMI (kg/m^2^), and age (yr). *D*: gene-set enrichment analysis of DMCs with increased or (*E*) decreased methylation intensity in AA relative to CA samples, showing “Enrichment,” or the number of genes containing at least one DMC relative to total number of genes in the pathway. Significance of DMCs was based on *P* < 0.05 and |Methylation difference| > 5%. For pathway enrichment, significance was weighted based on a Fisher’s Exact test.

To better understand the racial differences in cardiac DNA methylation in the testing cohort, a differential methylation analysis was performed comparing site-specific methylation of AA and CA samples. This analysis revealed 61,579 differentially methylated CpG-probes (DMCs, *P* < 0.05) corresponding with 17,686 known genes (Fig. 5*A*, Supplemental Table S1). Although the greatest proportion of DMCs (44.8%) was found to occur in gene coding regions, a disproportionate enrichment of DMCs (18.2%) was discovered within promoter-associated CpG islands (CGIs) relative to the EPIC array’s configuration (Fig. 5*B*); a much lower enrichment was seen among CGIs located within gene bodies (9.7%). Hierarchical clustering and heatmap visualization of the 15,516 promoter-associated DMCs found within CGIs (Supplemental Table S2) revealed a prominent, bidirectional signature of race-based differences in CpG methylation intensity (Fig. 5*C*). Corresponding gene-set enrichment analysis (GSEA) of DMCs identified a disproportionate hypermethylation of two metabolic pathways: “Type II diabetes mellitus” (*P* = 0.002, 13% enriched) and “Fatty acid biosynthesis” (*P* = 0.006, 23% enriched) (Fig. 5*D*). Conversely, pathways representing hypomethylated DMCs were more associated with immunologic and inflammatory phenotypes (Fig. 5*E*): “Phagosome” (*P* = 0.0002, 11.8% enriched), “Th1/Th2 cell differentiation” (*P* = 0.0002, 14.1% enriched), and “cell adhesion molecules” (*P* = 0.0003, 11.7% enriched). Taken together, these findings suggest that racial differences in cardiac DNA methylation exist within promoter-associated CGIs of genes associated with both established and novel pathways of cardiac dysfunction.

### Transcriptome-Wide Cardiac Gene Expression

**Figure 6.**
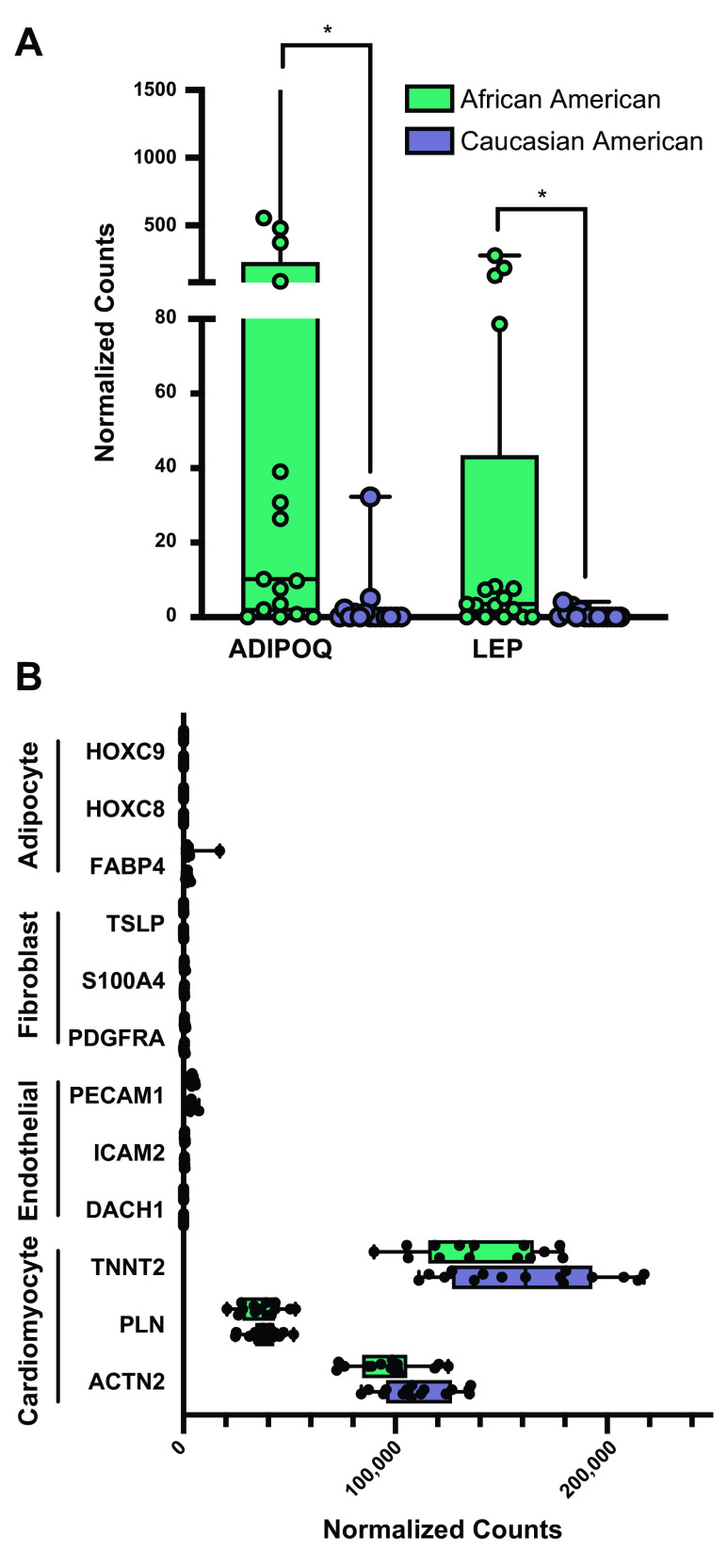
Transcriptomic analysis identified racial differences in adipokines. *A*: differential gene expression of adiponectin (ADIPOQ) and leptin (LEP) in African American (AA) compared with Caucasian American (CA) heart samples. *B*: gene markers of principal cell types in cardiac tissue, denoting expression in AA and CA heart samples. Statistical significance of AA relative to CA gene expression was based on multiple *t* test comparison (*P < 0.05).

To determine whether the signature of cardiac DNA methylation reflects differences in cardiac gene expression, we performed next-generation RNA sequencing of all cardiac samples. In contrast to our epigenomic analysis, unsupervised MDS failed to denote any separation by race (Supplemental Fig. S5). Differential expression by patient race identified 173 genes meeting Benjamini–Hochberg (BH)-adjusted *P* value significance (DEGs) (Supplemental Table S3), with pathway enrichment identifying “lung fibrosis” (*P* = 0.002), “adipokine signaling” (*P* = 0.003), and “TNF-related induction of apoptosis” (*P* = 0.006) as the three most-enriched pathways (Supplemental Table S4). Examination of the genes responsible for the enrichment of adipokine signaling revealed robustly elevated expression of adiponectin (ADIPOQ, *Q* = 0.04, 21.7-fold higher) and leptin (LEP, *Q* = 0.04, 11.2-fold higher) in AA samples relative to CA (Fig. 6*A*).

Variable tissue composition has been shown to influence differential expression analysis (31, 32). Therefore, to assess the potential for racial differences in tissue composition, we used cardiomyocyte (TNNT2, ACTN2, PLN), fibroblast (TSLP, S100A4, PDGFRA), endothelial cell (PECAM1, DACH1, ICAM2), and adipocyte-specific (HOXC8, HOXC9, FABP4) gene markers (19, 33–35) (Fig. 6*B*). This exposed a high degree of cardiomyocyte gene marker expression relative to other cell type markers, and no racial differences were seen. Due to the documented importance of immune cell composition on cardiac phenotype (36, 37), in silico method of cellular deconvolution was performed using CIBERSORT (38) to infer relative enrichment of immunologic cell types in our cardiac samples (Supplemental Fig. S6); again, no racial differences were noted. Therefore, gene expression analysis is unlikely confounded by cardiac tissue composition.

### Combined Epigenome-Transcriptome Analysis

**Figure 7.**
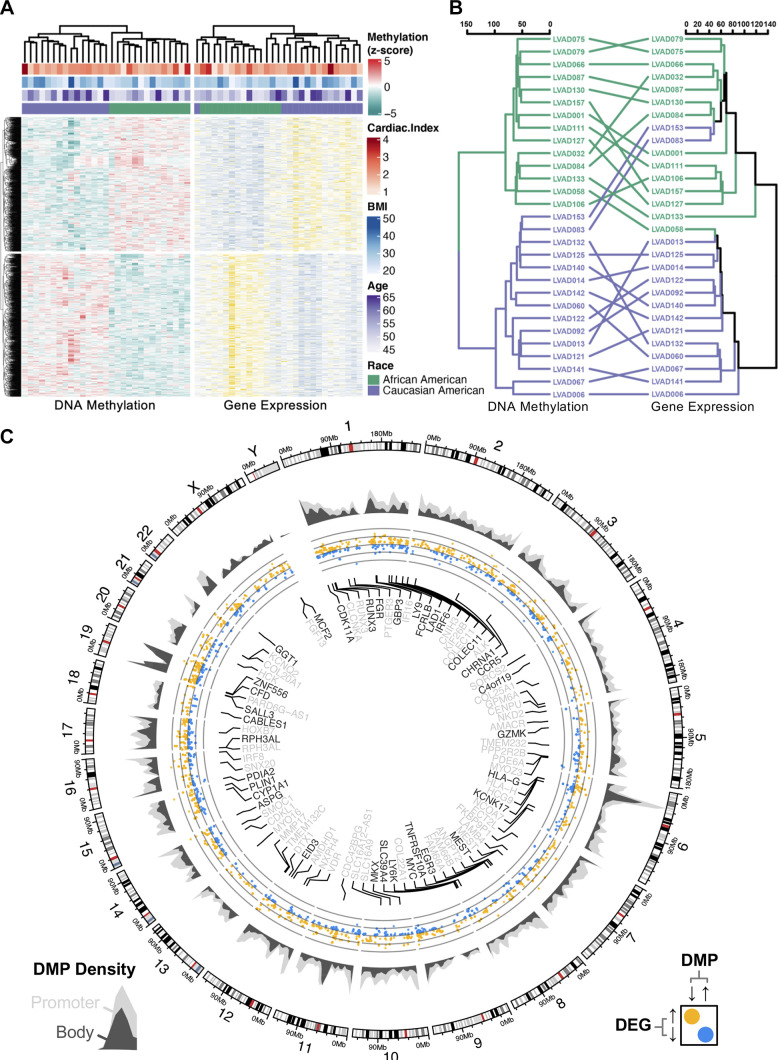
Combined cardiac transcriptome-methylome analysis. *A*: combination heatmap visualizing hierarchical clustering via squared Euclidean distance of promoter-associated differentially methylated CpG-probes (DMCs) found within the cytosine-p-guanine (CpG) islands of differentially expressed genes (DEGs). *B*: co-clustering analysis via “tanglegram” to compare the degree of “entanglement” between hierarchical clustering via Pearson’s correlation of both DMCs and DEGs; entanglement = 0.27 (0 = perfect alignment; 1 = no alignment). *C*: circular genome plot illustrating genomic distribution of DMCs annotating to promoter (light gray) and gene body (dark gray) regions. All DEGs were plotted based on log2(fold-difference) between African American (AA) and Caucasian American (CA) samples (yellow = higher in AA, blue = lower in AA). Fifty most-significant DEGs by *Q*-value were labeled according on their association with gene body or promoter methylation. *P* < 0.05.

Although no racial difference in transcriptome-wide cardiac gene expression was found, we sought to understand the relationship between promoter methylation and corresponding gene expression. We first examined the interrelationship between hierarchical clustering of genes possessing differentially methylated promoters (Fig. 7*A*). Owing to the inverse association between promoter methylation and gene expression (39, 40), we inspected DEGs possessing inversely methylated promoters. Among the 15,516 promoter-associated CpGs found to be differentially methylated between AA and CA failing hearts, 857 corresponded to inverse expression of an adjacent gene (Supplemental Table S5). To compare the relative similarity between dendrograms of DMCs and DEGs, a relative “entanglement” of 0.27 was computed between the DEG and DMC dendrograms (Fig. 7*B*). Genome-wide distribution of promoter and gene body DNA methylation densities was visualized, demonstrating regions enriched with differential DNA promoter—and gene body—methylation with concurrent gene expression differences (Fig. 7*C*). Pathway analysis of inverse differences in mRNA and promoter methylation enriched “Adipogenesis” (*P* = 0.003), “Nuclear Receptor Signaling” (*P* = 0.009), “NRF2 Signaling” (*P* = 0.02), “T Cell Antigen Receptor Signaling” (*P* = 0.03), and “ErbB Signaling” (*P* = 0.03) (Table 1). Notably, all genes responsible for driving the enrichment of the “Adipogenesis” were expressed at higher levels in AA relative to CA samples (refer to Supplemental Table S5).

**Table 1.** Enrichment analysis of inverse differences in gene expression and promoter methylation intensity

Enriched Pathway	Enrichment	*P* Value	Genes
Adipogenesis	5/130	0.003	CFD; CYP26B1; GATA3; PLIN1; SCD
Nuclear receptor pathway	7/319	0.009	CYP1A1; GGT1; MYC; NRG1; SCD; *SLC39A4*; *TGFA*
NRF2 signaling	4/146	0.02	GGT1; NRG1; *SLC39A4*; *TGFA*
T-cell antigen receptor signaling	3/90	0.03	CCR5; GATA3; LCP2
ErbB signaling	3/91	0.03	MYC; NRG1; *TGFA*

Pathways were sorted by Fisher’s exact test *P* value. Gene names formatted according to directionality of gene expression and methylation differences (bold = ↑DEGs, ↓DMCs; *underline and italics* = ↓DEGs, ↑DMCs). DEG, differentially expressed gene; DMC, differentially methylated CpG-probe.

### Geocoding for Socioeconomic Differences

**Figure 8.**
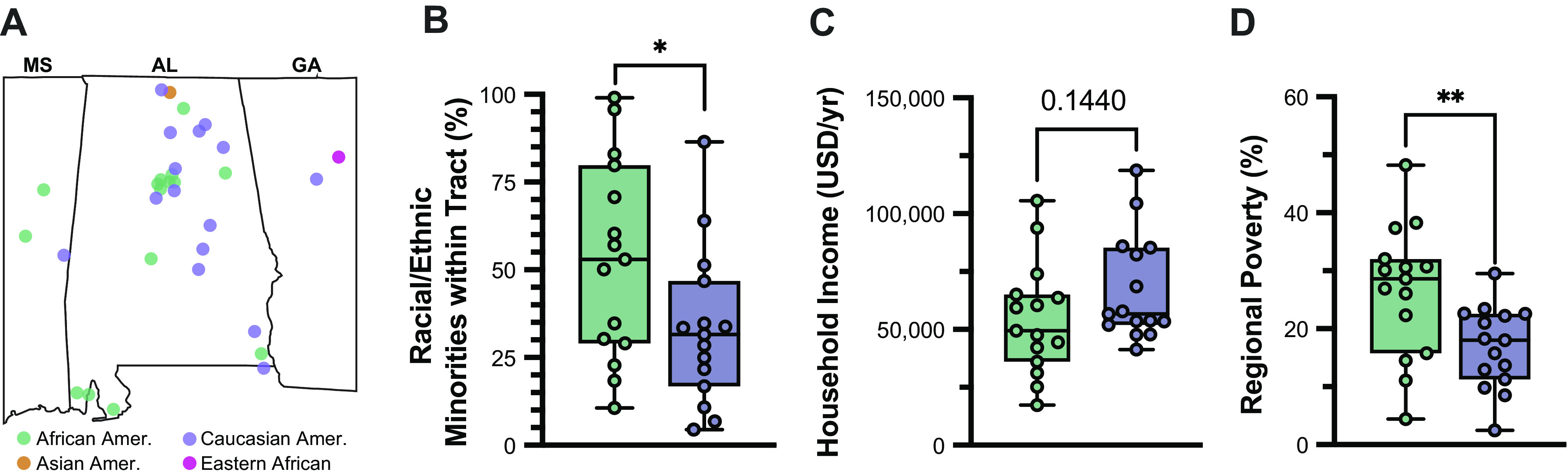
Geographical inference identifies racial disparity in end-stage heart failure (HF). *A*: FFEIC 2020 census data were used to infer income level based on census tract data from each patient, including (*B*) proportion of census constituents identifying with a racial or ethnic minority, (*C*) estimated family income (USD/yr), and (*D*) percentage of census falling below the federal poverty line. Statistical significance was based on unpaired Student’s *t* test (*P < 0.05; **P < 0.01). Graphs illustrate the interquartile range (box) and 95% confidence interval (error bars).

Despite the racially delineated differences noted in our analysis of cardiac DNA methylation, race is not a biological trait, but rather a crude generalization that is often accompanied by a complex social framework of community values, experiences, heritage, and geography (41); shared physical traits are merely an association with social factors (42). Therefore, our goal became to identify factors associated with self-reported race in cardiac DNA methylation. As stated, patient records lacked clinical evidence of racial differences (see Fig. 2), so we employed an indirect method of inferring demographic information based on geographical residence, as previously reported (43). Five-digit zip codes from subjects’ area of residence were matched to corresponding geographical tracts based on the 2020 Census survey (Fig. 8*A*). The Federal Financial Institutions Examination Council (FFIEC) geocoding system was then used to parse tract-based estimations of annual family income, poverty, and racial or ethnic population. From this, it was noted that AA subjects lived within census tracts of greater racial diversity (10.7%–99.1% minority population), whereas CA subjects resided in regions containing a lower composition of racial/ethnic minorities (Fig. 8*B*). Although gross family income was not significantly different by race (Fig. 8*C*), census tracts of AA contained disproportionately higher poverty density relative to those of CA subjects (Fig. 8*D*). Consistent with prior reports (44), these observations collectively support that racist geographical segregation exists within our patient cohort despite otherwise indistinguishable medical records before LVAD.

## DISCUSSION

As the clinical end point of numerous genetic and environmental conditions, HF constitutes a multifaceted and often intractable syndrome (45, 46). Owing to its widespread clinical heterogeneity that impedes the development of effective therapies for HF (8), the current multi-cohort study sought to identify the distinct features of end-stage HF that reflect differences in the cardiac epigenome. Using a genome-wide approach to develop signatures of cardiac DNA methylation, we discovered a bimodal distribution of CpG methylation intensity among the most-variable CpG loci. Many patient characteristics and comorbidities were superimposed, but only self-identified patient race could delineate this epigenetic pattern.

As also found in our patient cohort, African Americans experience worse clinical outcomes than any US race or ethnicity, even after adjusting for traditional risk factors (25, 47). Although HF is a common reason for hospitalization nationwide (48), AAs with HF are hospitalized at a rate 2.5-times higher than other races/ethnicities (49). Furthermore, despite a threefold higher mortality from HF complications (50), the prevalence of HF among AAs continues to increase, and it is estimated that 3.6% of this community will live with HF by 2030, exceeding the predicted prevalence of any other race/ethnicity in the US (51). It is therefore paramount to both identify and address the issues that underlie these disturbing racial differences in HF morbidity and mortality.

Although we identified a racial pattern of cardiac DNA methylation of end-stage HF, careful interpretation of this discovery is needed. Despite numerous epidemiologic reports of racial health disparities in HF, race is itself a poor proxy of disease physiology (52). Black communities indeed face a higher risk of cardiovascular disease (53, 54), and a higher prevalence of traditional risk factors for HF is also seen, such as hypertension (55), diabetes mellitus (56), obesity (57), and atherosclerosis (58). However, unlike other surrogates of disease susceptibility, which can be identified and modified, race is a dynamic social construct that encompasses cultural, demographic, regional, and ancestral backgrounds (41). Therefore, it is both imprudent and, frankly, unethical to use race as a clinical surrogate of disease susceptibility without also pursuing the underlying driver(s) of racial health differences. Moreover, the use of race as a clinical proxy has been publicly refuted as a practice that could perpetuate implicit racism in the healthcare setting (42).

For this reason, we searched for the underlying factor(s) responsible for racial differences in cardiac DNA methylation of our two cohorts. Unfortunately, further review of patients’ medical records exposed only the realization that social and demographic factors are rarely documented. By contrast, complex social constructs such as race and gender are rarely absent, though these metrics offer little insight into the physiological underpinnings of chronic disease. Although our geographical coding analysis provided indirect evidence of socioeconomic inequity, we hope to stress the importance of thorough and up-to-date clinical documentation of social and environmental factors in patient encounters. Doing so will likely provide the social context needed to better understand underlying determinants of each patient’s disease.

Nevertheless, the existence of genetic, behavioral, and environmental contributions to racial determinants of health should not be entirely disregarded. As a biomarker of HF, N-terminal pro B-type natriuretic peptide (NTproBNP) has been found to underestimate the severity of HF decompensation in AAs (59–63). As a protein synthesized by cardiomyocytes in response to ventricular stretch, NTproBNP stimulates diuresis and a subsequent reduction in circulatory demand (64, 65). This protein was first identified as HF biomarker using a largely white cohort that lacked sufficient representation from other racial and ethnic population (66, 67). Since its widespread clinical use, AAs have been found to exhibit lower baseline levels of plasma NTproBNP than do CA patients (68). The differential expression of noncoding RNAs, specifically microRNA-425, has been implicated as one possible explanation (68).

Our analysis identified differential promoter methylation of genes involved in fatty acid metabolism among AA relative to CA samples, along with higher expression of lipogenesis genes including perilipin-1 (PLN1), adiponectin (ADIPOQ), and leptin (LEP). Metabolic perturbations are a pathological hallmark of end-stage HF (69), wherein the heart becomes more glycolytic when it fails. This metabolic switch has been found to occur even before manifestations of cardiac dysfunction (70), and is sufficient to impair cardiac function (70–72). Despite mechanical circulatory support via LVAD implantation, the heart persistently suppresses fatty acid oxidation (73). In a subset of patients with HF, however, myocardial “reverse remodeling” has been observed in response to LVAD-induced cardiac unloading (74, 75), which is accompanied by metabolic reversal to a non-failing phenotype (76). Therefore, we hypothesize that the epigenetic remodeling of cardiac metabolism determines the therapeutic potential of LVADs. Our group has recently shown that promoter methylation accompanies the cardiac metabolic reprogramming that occurs in both ischemic and dilated etiologies of human HF (18, 19). Therefore, we hypothesize that epigenetic reprogramming of cardiac metabolism constitutes a pathological mechanism that may influence responsiveness to LVAD-induced cardiac unloading.

Conversely, genes found to exhibit differentially hypomethylated promoters in AA hearts disproportionately represented inflammatory signaling cascades. Preliminary reports provide evidence of low-grade inflammation among children of African American and Hispanic communities (77). This observation has been cited in association with an epidemiologic phenomenon termed “weathering,” wherein chronic stressors associated with racial and/or socioeconomic discrimination could accelerate the onset of cardiovascular disease (78). In light of the coronavirus pandemic, recent studies have identified individuals of African descent as nearly twice as likely than those of European descent to both contract and die from SARS-CoV-2, the virus responsible for the COVID-19 pandemic (79, 80). Cardiovascular complications are among the main reasons for this higher mortality (81), with the disproportionate number developing prothrombotic and proinflammatory “cytokine storming” (82). It is therefore possible—though untested—that early life stresses associated with racial and socioeconomic discrimination predispose the heart to a more proinflammatory mode of cardiac injury and failure.

Owing to both the observational nature and limited sample size of our current study, several unanswered questions remain. First, our analysis was developed using a tissue database that lacked sufficient samples from female patients, resulting in our focus on exclusively male patients; future studies of the cardiac epigenome must therefore consider both sex and gender as likely sources of heterogeneity, as previously reported (49, 83). Since we were not able to analyze cardiac DNA methylation in “healthy” individuals, we cannot determine whether these racial and socioeconomic differences in cardiac DNA methylation are exclusively present among patients with end-stage heart failure. Although our use of LVAD cores permits the analysis of human cardiac tissue, a stringent interdisciplinary review must occur to ensure that patients undergoing LVAD implantation possess reliable social and financial infrastructure necessary to benefit from outpatient mechanical support. Although our analysis revealed socioeconomic disparity, the samples obtained from patients receiving LVAD implantation unlikely reflect the socioeconomic and clinical diversity present in the HF patient population. Our use of multiple cohorts strengthens our finding of distinct methylation signatures, but larger multi-institutional studies are needed to make population-wide inferences. Lastly, despite our use of computational methods to estimate and minimize genetic contributions, it is nevertheless possible that genetic background may influence our findings.

### Conclusions

In this single-center study, we report that racial dissimilarities in cardiac DNA methylation exist among patients with end-stage heart failure. Racial differences were significantly confounded by socioeconomic disparity, which likely influence differential susceptibility to cardiovascular disease. Greater emphasis should therefore be placed upon clinicians to identify, document, and address these social contributors of HF susceptibility. Such an effort would facilitate patient-centered studies that could unlock the therapeutic potential of cardiac epigenomics. Nevertheless, in the present study, we provide preliminary evidence that socioeconomic factors are likely responsible for racial differences in cardiac DNA methylation among men with end-stage heart failure.

## GRANTS

Financial support for this work was provided to A. R. Wende by pilot awards from the Center for Healthy African American Men through Partnerships (CHAAMPS) NIH U54 MD008620 as well as the UAB Center for Clinical and Translational Science (CCTS) NIH UL1 TR001417 and NIH National Heart, Lung, and Blood Institute R01 HL133011. Training support was provided to M. E. Pepin by both an NIH predoctoral F30 HL137240 and a postdoctoral fellowship from the Alexander von Humboldt foundation. L. A. Potter is supported by an NIH predoctoral F31 HL154571. B. A. Hidalgo was supported by NIH K01 HL130609 01 and an American Heart Association Precision Medicine Institute Grant.

## DISCLOSURES

No conflicts of interest, financial or otherwise, are declared by the authors.

## AUTHOR CONTRIBUTIONS

A.R.W. conceived and designed research; C-M.H. performed experiments; M.E.P., L.A.P., S.B., J.P.B., A.H.A., S.V.P., S.M.V., and A.R.W. analyzed data; M.E.P., L.A.P., S.B., B.A.H., and A.R.W. interpreted results of experiments; M.E.P., L.A.P., and S.B. prepared figures; M.E.P. drafted manuscript; M.E.P., C-M.H., L.A.P., S.B., S.M.P., B.A.H., and A.R.W. edited and revised manuscript; M.E.P., C-M.H., L.A.P., S.B., J.P.B., A.H.A., S.M.P., S.V.P., B.A.H., S.M.V., and A.R.W. approved final version of manuscript.

## References

[1] Papadimitriou L , Moore CK , Butler J , Long RC . The limitations of symptom-based heart failure management. Card Fail Rev 5: 74–77, 2019. doi:10.15420/cfr.2019.3.2. 31179015 PMC6546002

[2] Buddeke J , Valstar GB , van Dis I , Visseren FLJ , Rutten FH , den Ruijter HM , Vaartjes I , Bots ML ; Queen of Heart and RECONNECT Investigators. Mortality after hospital admission for heart failure: improvement over time, equally strong in women as in men. BMC Public Health 20: 36, 2020. doi:10.1186/s12889-019-7934-3. 31924185 PMC6954619

[3] Sahli Costabal F , Choy JS , Sack KL , Guccione JM , Kassab GS , Kuhl E . Multiscale characterization of heart failure. Acta Biomater 86: 66–76, 2019. doi:10.1016/j.actbio.2018.12.053. 30630123 PMC6369012

[4] Czepluch FS , Wollnik B , Hasenfuß G . Genetic determinants of heart failure: facts and numbers. ESC Heart Fail 5: 211–217, 2018. doi:10.1002/ehf2.12267. 29457878 PMC5933969

[5] Haas J , Frese KS , Peil B , Kloos W , Keller A , Nietsch R , Feng Z , , et al. Atlas of the clinical genetics of human dilated cardiomyopathy. Eur Heart J 36: 1123–1135, 2015. doi:10.1093/eurheartj/ehu301. 25163546

[6] Hershberger RE , Givertz MM , Ho CY , Judge DP , Kantor PF , McBride KL , Morales A , Taylor MRG , Vatta M , Ware SM . Genetic evaluation of cardiomyopathy—a Heart Failure Society of America practice guideline. J Card Fail 24: 281–302, 2018. doi:10.1016/j.cardfail.2018.03.004. 29567486 PMC9903357

[7] Petretta M , Pirozzi F , Sasso L , Paglia A , Bonaduce D . Review and metaanalysis of the frequency of familial dilated cardiomyopathy. Am J Cardiol 108: 1171–1176, 2011. doi:10.1016/j.amjcard.2011.06.022. 21798502

[8] Lund LH . The inescapable heterogeneity of heart failure. J Card Fail 23: 351–352, 2017. doi:10.1016/j.cardfail.2017.03.007. 28344108

[9] Cosselman KE , Navas-Acien A , Kaufman JD . Environmental factors in cardiovascular disease. Nat Rev Cardiol 12: 627–642, 2015. doi:10.1038/nrcardio.2015.152. 26461967

[10] Wolk MJ , Scheidt S , Killip T . Heart failure complicating acute myocardial infarction. Circulation 45: 1125–1138, 1972. doi:10.1161/01.cir.45.5.1125. 5020802

[11] Rodeheffer RJ . Hypertension and heart failure: the ALLHAT imperative. Circulation 124: 1803–1805, 2011. doi:10.1161/CIRCULATIONAHA.111.059303. 22025634

[12] Kenny HC , Abel ED . Heart failure in type 2 diabetes mellitus. Circ Res 124: 121–141, 2019. doi:10.1161/CIRCRESAHA.118.311371. 30605420 PMC6447311

[13] Riegel B , Moser DK , Powell M , Rector TS , Havranek EP . Nonpharmacologic care by heart failure experts. J Card Fail 12: 149–153, 2006. doi:10.1016/j.cardfail.2005.10.004. 16520265

[14] Vujic A , Lerchenmuller C , Wu TD , Guillermier C , Rabolli CP , Gonzalez E , Senyo SE , Liu X , Guerquin-Kern JL , Steinhauser ML , Lee RT , Rosenzweig A . Exercise induces new cardiomyocyte generation in the adult mammalian heart. Nat Commun 9: 1659, 2018. doi:10.1038/s41467-018-04083-1. 29695718 PMC5916892

[15] Shah AS , Langrish JP , Nair H , McAllister DA , Hunter AL , Donaldson K , Newby DE , Mills NL . Global association of air pollution and heart failure: a systematic review and meta-analysis. Lancet 382: 1039–1048, 2013. doi:10.1016/s0140-6736(13)60898-3. 23849322 PMC3809511

[16] Arab S , Liu PP . Heart failure in the post-genomics era: gene-environment interactions. Curr Opin Mol Ther 7: 577–582, 2005. 16370381

[17] Gillette TG , Hill JA . Readers, writers, and erasers: chromatin as the whiteboard of heart disease. Circ Res 116: 1245–1253, 2015. doi:10.1161/CIRCRESAHA.116.303630. 25814685 PMC4380191

[18] Pepin ME , Drakos S , Ha CM , Tristani-Firouzi M , Selzman CH , Fang JC , Wende AR , Wever-Pinzon O . DNA methylation reprograms cardiac metabolic gene expression in end-stage human heart failure. Am J Physiol Heart Circ Physiol 317: H674–H684, 2019. doi:10.1152/ajpheart.00016.2019. 31298559 PMC6843013

[19] Pepin ME , Ha CM , Crossman DK , Litovsky SH , Varambally S , Barchue JP , Pamboukian SV , Diakos NA , Drakos SG , Pogwizd SM , Wende AR . Genome-wide DNA methylation encodes cardiac transcriptional reprogramming in human ischemic heart failure. Lab Invest 99: 371–386, 2019. doi:10.1038/s41374-018-0104-x. 30089854 PMC6515060

[20] Sharma S , Razeghi P , Shakir A , Keneson BJ 2nd, Clubb F , Taegtmeyer H . Regional heterogeneity in gene expression profiles: a transcript analysis in human and rat heart. Cardiology 100: 73–79, 2003. doi:10.1159/000073042. 14557693

[21] Maksimovic J , Gordon L , Oshlack A . SWAN: subset-quantile within array normalization for illumina infinium human methylation 450 BeadChips. Genome Biol 13: R44, 2012. doi:10.1186/gb-2012-13-6-r44. 22703947 PMC3446316

[22] Touleimat N , Tost J . Complete pipeline for infinium® human methylation 450K BeadChip data processing using subset quantile normalization for accurate DNA methylation estimation. Epigenomics 4: 325–341, 2012. doi:10.2217/epi.12.21. 22690668

[23] Smyth GK . Linear models and empirical bayes methods for assessing differential expression in microarray experiments. Stat Appl Genet Mol Biol 3: Article 3, 2004. doi:10.2202/1544-6115.1027. 16646809

[24] Love MI , Huber W , Anders S . Moderated estimation of fold change and dispersion for RNA-seq data with DESeq2. Genome Biol 15: 550, 2014. doi:10.1186/s13059-014-0550-8. 25516281 PMC4302049

[25] Okoh AK , Selevanny M , Singh S , Hirji S , Singh S , Al Obaidi N , Lee LY , Camacho M , Russo MJ . Racial disparities and outcomes of left ventricular assist device implantation as a bridge to heart transplantation. Esc Heart Fail 7: 2744–2751, 2020. doi:10.1002/ehf2.12866. 32627939 PMC7524221

[26] Hinoue T , Weisenberger DJ , Lange CP , Shen H , Byun HM , Van Den Berg D , Malik S , Pan F , Noushmehr H , van Dijk CM , Tollenaar RA , Laird PW . Genome-scale analysis of aberrant DNA methylation in colorectal cancer. Genome Res 22: 271–282, 2012. doi:10.1101/gr.117523.110. 21659424 PMC3266034

[27] Laffaire J , Everhard S , Idbaih A , Criniere E , Marie Y , de Reynies A , Schiappa R , Mokhtari K , Hoang-Xuan K , Sanson M , Delattre JY , Thillet J , Ducray F . Methylation profiling identifies 2 groups of gliomas according to their tumorigenesis. Neuro Oncol 13: 84–98, 2011. doi:10.1093/neuonc/noq110. 20926426 PMC3018904

[28] Noushmehr H , Weisenberger DJ , Diefes K , Phillips HS , Pujara K , Berman BP , Pan F , Pelloski CE , Sulman EP , Bhat KP , Verhaak RG , Hoadley KA , Hayes DN , Perou CM , Schmidt HK , Ding L , Wilson RK , Van Den Berg D , Shen H , Bengtsson H , Neuvial P , Cope LM , Buckley J , Herman JG , Baylin SB , Laird PW , Aldape K , Cancer Genome Atlas Research N ; Cancer Genome Atlas Research Network. Identification of a CpG island methylator phenotype that defines a distinct subgroup of glioma. Cancer Cell 17: 510–522, 2010. doi:10.1016/j.ccr.2010.03.017. 20399149 PMC2872684

[29] Sherry ST , Ward MH , Kholodov M , Baker J , Phan L , Smigielski EM , Sirotkin K . dbSNP: the NCBI database of genetic variation. Nucleic Acids Res 29: 308–311, 2001. doi:10.1093/nar/29.1.308. 11125122 PMC29783

[30] LaBarre BA , Goncearenco A , Petrykowska HM , Jaratlerdsiri W , Bornman MSR , Hayes VM , Elnitski L . MethylToSNP: identifying SNPs in Illumina DNA methylation array data. Epigenetics Chromatin 12: 79, 2019. doi:10.1186/s13072-019-0321-6. 31861999 PMC6923858

[31] Aran D , Hu Z , Butte AJ . xCell: digitally portraying the tissue cellular heterogeneity landscape. Genome Biol 18: 220, 2017. doi:10.1186/s13059-017-1349-1. 29141660 PMC5688663

[32] Shen-Orr SS , Gaujoux R . Computational deconvolution: extracting cell type-specific information from heterogeneous samples. Curr Opin Immunol 25: 571–578, 2013. doi:10.1016/j.coi.2013.09.015. 24148234 PMC3874291

[33] Forough R , Scarcello C , Perkins M . Cardiac biomarkers: a focus on cardiac regeneration. J Tehran Heart Cent 6: 179–186, 2011. 23074366 PMC3467959

[34] Ivey MJ , Tallquist MD . Defining the cardiac fibroblast. Circ J 80: 2269–2276, 2016. doi:10.1253/circj.CJ-16-1003. 27746422 PMC5588900

[35] Pinto AR , Ilinykh A , Ivey MJ , Kuwabara JT , D’Antoni ML , Debuque R , Chandran A , Wang L , Arora K , Rosenthal NA , Tallquist MD . Revisiting cardiac cellular composition. Circ Res 118: 400–409, 2016. doi:10.1161/CIRCRESAHA.115.307778. 26635390 PMC4744092

[36] Frieler RA , Mortensen RM . Immune cell and other noncardiomyocyte regulation of cardiac hypertrophy and remodeling. Circulation 131: 1019–1030, 2015. doi:10.1161/CIRCULATIONAHA.114.008788. 25779542 PMC4367123

[37] Patel B , Bansal SS , Ismahil MA , Hamid T , Rokosh G , Mack M , Prabhu SD . CCR2^+^ monocyte-derived infiltrating macrophages are required for adverse cardiac remodeling during pressure overload. JACC Basic Transl Sci 3: 230–244, 2018. doi:10.1016/j.jacbts.2017.12.006. 30062209 PMC6059350

[38] Kawada JI , Takeuchi S , Imai H , Okumura T , Horiba K , Suzuki T , Torii Y , Yasuda K , Imanaka-Yoshida K , Ito Y . Immune cell infiltration landscapes in pediatric acute myocarditis analyzed by CIBERSORT. J Cardiol 77: 174–178, 2021. doi:10.1016/j.jjcc.2020.08.004. 32891480

[39] Bird AP . CpG-rich islands and the function of DNA methylation. Nature 321: 209–213, 1986. doi:10.1038/321209a0. 2423876

[40] Jjingo D , Conley AB , Yi SV , Lunyak VV , Jordan IK . On the presence and role of human gene-body DNA methylation. Oncotarget 3: 462–474, 2012. doi:10.18632/oncotarget.497. 22577155 PMC3380580

[41] Loue S . Assessing Race, Ethnicity and Gender in Health. New York: Springer Sciences-Business Media, 2006, p. xv, 158. doi:10.1007/978-0-387-32462-3.

[42] Obermeyer Z , Powers B , Vogeli C , Mullainathan S . Dissecting racial bias in an algorithm used to manage the health of populations. Science 366: 447–453, 2019. doi:10.1126/science.aax2342. 31649194

[43] Prager M , Kurz C , Bohm J , Laxy M , Maier W . Using data from online geocoding services for the assessment of environmental obesogenic factors: a feasibility study. Int J Health Geogr 18: 13, 2019. doi:10.1186/s12942-019-0177-9. 31174531 PMC6555943

[44] Bucholz EM , Ma S , Normand SL , Krumholz HM . Race, socioeconomic status, and life expectancy after acute myocardial infarction. Circulation 132: 1338–1346, 2015. doi:10.1161/CIRCULATIONAHA.115.017009. 26369354 PMC5097251

[45] Kao D , Purohit S , Jhund P . Therapeutic futility and phenotypic heterogeneity in heart failure with preserved ejection fraction: what is the role of bionic learning? Eur J Heart Fail 22: 159–161, 2020. doi:10.1002/ejhf.1658. 31749260 PMC7301725

[46] Khan MS , Li L , Yasmin F , Khan SU , Bajaj NS , Pandey A , Murad MH , Fonarow GC , Butler J , Vaduganathan M . Assessment of heterogeneity in heart failure-related meta-analyses. Circ Heart Fail 13: e007070, 2020. doi:10.1161/circheartfailure.120.007070. 33131285

[47] Virani SS , Alonso A , Benjamin EJ , Bittencourt MS , Callaway CW , Carson AP , , et al.; American Heart Association Council on Epidemiology, Prevention Statistics Commitee, and Stroke Statistics Subcommitee. Heart disease and stroke statistics—2020 update: a report from the American Heart Association. Circulation 141: e139–e596, 2020. doi:10.1161/CIR.0000000000000757. 31992061

[48] Jackson SL , Tong X , King RJ , Loustalot F , Hong Y , Ritchey MD . National burden of heart failure events in the United States, 2006 to 2014. Circ Heart Fail 11: e004873, 2018. doi:10.1161/circheartfailure.117.004873. 30562099 PMC6424109

[49] Ziaeian B , Kominski GF , Ong MK , Mays VM , Brook RH , Fonarow GC . National differences in trends for heart failure hospitalizations by sex and race/ethnicity. Circ Cardiovasc Qual Outcomes 10: e003552, 2017. doi:10.1161/CIRCOUTCOMES.116.003552. 28655709 PMC5540644

[50] Glynn P , Lloyd-Jones DM , Feinstein MJ , Carnethon M , Khan SS . Disparities in cardiovascular mortality related to heart failure in the United States. J Am Coll Cardiol 73: 2354–2355, 2019. doi:10.1016/j.jacc.2019.02.042. 31072580

[51] Heidenreich PA , Albert NM , Allen LA , Bluemke DA , Butler J , Fonarow GC , Ikonomidis JS , Khavjou O , Konstam MA , Maddox TM , Nichol G , Pham M , Pina IL , Trogdon JG ; American Heart Association Advocacy Coordinating Commitee, Council on Arteriosclerosis Thrombosis and Vascular Biology, Council on Cardiovascular Radiology and Intervention, Council on Clinical Cardiology, Council on Epidemiology and Prevention, and Stroke Council. Forecasting the impact of heart failure in the United States: a policy statement from the American Heart Association. Circ Heart Fail 6: 606–619, 2013. doi:10.1161/hhf.0b013e318291329a. 23616602 PMC3908895

[52] Wolf ST , Jablonski NG , Kenney WL . Examining “race” in physiology. Am J Physiol Heart Circ Physiol 319: H1409–H1413, 2020. doi:10.1152/ajpheart.00698.2020. 33064554 PMC7792710

[53] Kurian AK , Cardarelli KM . Racial and ethnic differences in cardiovascular disease risk factors: a systematic review. Ethn Dis 17: 143–152, 2007. 17274224

[54] Meadows TA , Bhatt DL , Cannon CP , Gersh BJ , Rother J , Goto S , Liau CS , Wilson PW , Salette G , Smith SC , Steg PG ; REACH Registry Investigators. Ethnic differences in cardiovascular risks and mortality in atherothrombotic disease: insights from the REduction of Atherothrombosis for Continued Health (REACH) registry. Mayo Clin Proc 86: 960–967, 2011. doi:10.4065/mcp.2011.0010. 21964173 PMC3184025

[55] Fei K , Rodriguez-Lopez JS , Ramos M , Islam N , Trinh-Shevrin C , Yi SS , Chernov C , Perlman SE , Thorpe LE . Racial and ethnic subgroup disparities in hypertension prevalence, New York City Health and Nutrition Examination Survey, 2013–2014. Prev Chronic Dis 14: E33, 2017. doi:10.5888/pcd14.160478. 28427484 PMC5420441

[56] Zhu Y , Sidell MA , Arterburn D , Daley MF , Desai J , Fitzpatrick SL , Horberg MA , Koebnick C , McCormick E , Oshiro C , Young DR , Ferrara A . Racial/ethnic disparities in the prevalence of diabetes and prediabetes by BMI: Patient Outcomes Research To Advance Learning (PORTAL) multisite cohort of adults in the U.S. Diabetes Care 42: 2211–2219, 2019. doi:10.2337/dc19-0532. 31537541 PMC6868463

[57] Petersen R , Pan L , Blanck HM . Racial and ethnic disparities in adult obesity in the United States: CDC’s tracking to inform state and local action. Prev Chronic Dis 16: E46, 2019. doi:10.5888/pcd16.180579. 30974071 PMC6464044

[58] Leigh JA , Alvarez M , Rodriguez CJ . Ethnic minorities and coronary heart disease: an update and future directions. Curr Atheroscler Rep 18: 9, 2016. doi:10.1007/s11883-016-0559-4. 26792015 PMC4828242

[59] Bajaj NS , Gutierrez OM , Arora G , Judd SE , Patel N , Bennett A , Prabhu SD , Howard G , Howard VJ , Cushman M , Arora P . Racial differences in plasma levels of n-terminal pro-b-type natriuretic peptide and outcomes: the Reasons for Geographic and Racial Differences in Stroke (REGARDS) Study. JAMA Cardiol 3: 11–17, 2018. doi:10.1001/jamacardio.2017.4207. 29167879 PMC5833525

[60] Gupta DK , Claggett B , Wells Q , Cheng S , Li M , Maruthur N , Selvin E , Coresh J , Konety S , Butler KR , Mosley T , Boerwinkle E , Hoogeveen R , Ballantyne CM , Solomon SD . Racial differences in circulating natriuretic peptide levels: the atherosclerosis risk in communities study. J Am Heart Assoc 4: e001831, 2015. doi:10.1161/jaha.115.001831. 25999400 PMC4599412

[61] Gupta DK , de Lemos JA , Ayers CR , Berry JD , Wang TJ . Racial differences in natriuretic peptide levels: the Dallas Heart Study. JACC Heart Fail 3: 513–519, 2015. doi:10.1016/j.jchf.2015.02.008. 26071618 PMC4498971

[62] Gupta DK , Walford GA , Ma Y , Jarolim P , Wang TJ , Group DPPR ; DPP Research Group. Racial/ethnic differences in circulating natriuretic peptide levels: the Diabetes Prevention Program. PLoS One 15: e0229280, 2020. doi:10.1371/journal.pone.0229280. 32084251 PMC7034896

[63] Krim SR , Vivo RP , Krim NR , Qian F , Cox M , Ventura H , Hernandez AF , Bhatt DL , Fonarow GC . Racial/ethnic differences in B-type natriuretic peptide levels and their association with care and outcomes among patients hospitalized with heart failure: findings from Get With The Guidelines-Heart Failure. JACC Heart Fail 1: 345–352, 2013. doi:10.1016/j.jchf.2013.04.008. 24621938

[64] Sudoh T , Kangawa K , Minamino N , Matsuo H . A new natriuretic peptide in porcine brain. Nature 332: 78–81, 1988. doi:10.1038/332078a0. 2964562

[65] Yasue H , Yoshimura M , Sumida H , Kikuta K , Kugiyama K , Jougasaki M , Ogawa H , Okumura K , Mukoyama M , Nakao K . Localization and mechanism of secretion of B-type natriuretic peptide in comparison with those of A-type natriuretic peptide in normal subjects and patients with heart failure. Circulation 90: 195–203, 1994. doi:10.1161/01.CIR.90.1.195. 8025996

[66] Januzzi JL , van Kimmenade R , Lainchbury J , Bayes-Genis A , Ordonez-Llanos J , Santalo-Bel M , Pinto YM , Richards M . NT-proBNP testing for diagnosis and short-term prognosis in acute destabilized heart failure: an international pooled analysis of 1256 patients: the International Collaborative of NT-proBNP Study. Eur Heart J 27: 330–337, 2006. doi:10.1093/eurheartj/ehi631. 16293638

[67] Moe GW , Howlett J , Januzzi JL , Zowall H ; Canadian Multicenter Improved Management of Patients With Congestive Heart Failure Study Investigators. N-terminal pro-B-type natriuretic peptide testing improves the management of patients with suspected acute heart failure: primary results of the Canadian prospective randomized multicenter IMPROVE-CHF study. Circulation 115: 3103–3110, 2007. doi:10.1161/circulationaha.106.666255. 17548729

[68] Patel N , Russell GK , Musunuru K , Gutierrez OM , Halade G , Kain V , Lv W , Prabhu SD , Margulies KB , Cappola TP , Arora G , Wang TJ , Arora P . Race, natriuretic peptides, and high-carbohydrate challenge: a clinical trial. Circ Res 125: 957–968, 2019. doi:10.1161/circresaha.119.315026. 31588864 PMC7033629

[69] Danforth WH , Ballard FB , Kako K , Choudhury JD , Bing RJ . Metabolism of the heart in failure. Circulation 21: 112–123, 1960. doi:10.1161/01.cir.21.1.112. 13813953

[70] Kundu BK , Zhong M , Sen S , Davogustto G , Keller SR , Taegtmeyer H . Remodeling of glucose metabolism precedes pressure overload-induced left ventricular hypertrophy: review of a hypothesis. Cardiology 130: 211–220, 2015. doi:10.1159/000369782. 25791172 PMC4394867

[71] Jebessa ZH , Shanmukha KD , Dewenter M , Lehmann LH , Xu C , Schreiter F , Siede D , Gong XM , Worst BC , Federico G , Sauer SW , Fischer T , Wechselberger L , Muller OJ , Sossalla S , Dieterich C , Most P , Grone HJ , Moro C , Oberer M , Haemmerle G , Katus HA , Tyedmers J , Backs J . The lipid droplet-associated protein ABHD5 protects the heart through proteolysis of HDAC4. Nat Metab 1: 1157–1167, 2019. doi:10.1038/s42255-019-0138-4. 31742248 PMC6861130

[72] Wende AR , Brahma MK , McGinnis GR , Young ME . Metabolic origins of heart failure. JACC Basic Transl Sci 2: 297–310, 2017. doi:10.1016/j.jacbts.2016.11.009. 28944310 PMC5609457

[73] Diakos NA , Navankasattusas S , Abel ED , Rutter J , McCreath L , Ferrin P , McKellar SH , Miller DV , Park SY , Richardson RS , Deberardinis R , Cox JE , Kfoury AG , Selzman CH , Stehlik J , Fang JC , Li DY , Drakos SG . Evidence of glycolysis up-regulation and pyruvate mitochondrial oxidation mismatch during mechanical unloading of the failing human heart: implications for cardiac reloading and conditioning. JACC Basic Transl Sci 1: 432–444, 2016. doi:10.1016/j.jacbts.2016.06.009. 28497127 PMC5422992

[74] Dipla K , Mattiello JA , Jeevanandam V , Houser SR , Margulies KB . Myocyte recovery after mechanical circulatory support in humans with end-stage heart failure. Circulation 97: 2316–2322, 1998. doi:10.1161/01.CIR.97.23.2316. 9639375

[75] Drakos SG , Wever-Pinzon O , Selzman CH , Gilbert EM , Alharethi R , Reid BB , Saidi A , Diakos NA , Stoker S , Davis ES , Movsesian M , Li DY , Stehlik J , Kfoury AG ; UCAR (Utah Cardiac Recovery Program) Investigators. Magnitude and time course of changes induced by continuous-flow left ventricular assist device unloading in chronic heart failure: insights into cardiac recovery. J Am Coll Cardiol 61: 1994–2013, 1985. doi:10.1016/j.jacc.2013.01.072. 23500219 PMC3819804

[76] Rame JE , Birks EJ . Metabolic reprogramming after left ventricular assist device: remodeling without recovery of cardiac energetics. JACC Basic Transl Sci 1: 445–448, 2016. doi:10.1016/j.jacbts.2016.09.003. 30167531 PMC6113417

[77] Schmeer KK , Tarrence J . Racial-ethnic disparities in inflammation: evidence of weathering in childhood? J Health Soc Behav 59: 411–428, 2018. doi:10.1177/0022146518784592. 29949724 PMC6177208

[78] Das A . How does race get “under the skin”?: inflammation, weathering, and metabolic problems in late life. Soc Sci Med 77: 75–83, 2013. doi:10.1016/j.socscimed.2012.11.007. 23201190 PMC3587959

[79] Hawkins D . Differential occupational risk for COVID-19 and other infection exposure according to race and ethnicity. Am J Ind Med 63: 817–820, 2020. doi:10.1002/ajim.23145. 32539166 PMC7323065

[80] Henning-Smith C , Tuttle M , Kozhimannil KB . Unequal distribution of COVID-19 risk among rural residents by race and ethnicity. J Rural Health 37: 224–226, 2021. doi:10.1111/jrh.12463. 32396220 PMC7273062

[81] Chin-Hong P , Alexander KM , Haynes N , Albert MA ; The Association of Black Cardiologists. Pulling at the heart: COVID-19, race/ethnicity and ongoing disparities. Nat Rev Cardiol 17: 533–535, 2020. doi:10.1038/s41569-020-0416-6. 32732989 PMC7640988

[82] McGonagle D , Plein S , O'Donnell JS , Sharif K , Bridgewood C . Increased cardiovascular mortality in African Americans with COVID-19. Lancet Respir Med 8: 649–651, 2020. doi:10.1016/S2213-2600(20)30244-7. 32473125 PMC7255150

[83] Eisenberg E , Di Palo KE , Pina IL . Sex differences in heart failure. Clin Cardiol 41: 211–216, 2018. doi:10.1002/clc.22917. 29485677 PMC6489832

